# Beyond dopamine: Broader neuromodulatory contributions in sign- and goal-tracking

**DOI:** 10.1007/s00213-026-07013-y

**Published:** 2026-03-04

**Authors:** Amber Datta, Arya Datta, Noah Collins, Stephanie L. Grella, Ali Gheidi

**Affiliations:** 1 Lincoln Memorial University-DeBusk College of Osteopathic Medicine, 6965 Cumberland Gap Pkwy, Harrogate, TN USA; 2 Mercer University School of Medicine, Macon, GA USA; 3 Department of Psychology, Program In Neuroscience, Loyola University Chicago, 1032 W Sheridan Rd, Chicago, IL USA

**Keywords:** Sign tracking, Goal tracking, Individual differences, Addiction

## Abstract

Sign- and goal-tracking behaviors provide a powerful framework for understanding individual differences in cue-motivated responding and vulnerability to addiction. While dopamine has long been recognized as central to the attribution of incentive salience, growing evidence indicates that multiple neuromodulatory systems contribute to these behaviors through distinct mechanisms. This review synthesizes findings on the cholinergic, serotonergic, adrenergic, opioid, and stimulant (nicotine, caffeine) systems in modulating sign- and goal-tracking in rodents. A better understanding of these mechanisms may guide individualized interventions to reduce cue-triggered relapse and improve addiction treatment outcomes.

## Why study individual differences

Understanding individual differences is critical for uncovering the neurobiological mechanisms that drive vulnerability to substance abuse disorders. While many individuals experiment with drugs, only a subset develops compulsive use, highlighting the need to investigate why some brains are more susceptible than others (Deroche-Gamonet et al. 2004; George and Koob 2017). Animal models play a key role in identifying both shared and divergent neurobiological pathways underlying substance use. Studying these individual differences not only reveals novel targets for intervention but also opens the door to more personalized therapeutic strategies that address the specific molecular or circuit-level alterations contributing to addiction risk (Ragia and Manolopoulos 2018; Yadid et al. 2013).

### Sign tracking as a model to understand individual differences in addiction

One well-established model of individual differences relevant to addiction is the distinction between sign-tracking and goal-tracking behavior (Gheidi et al. 2023; Robinson and Flagel 2009). In Pavlovian conditioning paradigms, some animals (sign-trackers, STs) (Hearst and Jenkins 1974) are drawn to cues that predict reward delivery, while others (goal-trackers, GTs) (Boakes 1977) focus on the location of reward availability. A third phenotype (intermediate trackers, ITs) exists along this continuum, which does not show dominant responding to one or the other, demonstrating a mixture of ST and GT with no clear preference for either. This suggests that there are fundamental differences in how incentive salience is attributed (Saunders and Robinson 2013). For instance, STs and GTs differ in how they respond to both cues predicting reward and the reward itself. Given the choice between cocaine and food, STs prefer cocaine more often than GTs (Tunstall and Kearns 2015), and ST behavior has been linked to impulsivity (Flagel et al. 2010; Lovic et al. 2011).

Pavlovian conditioned approach (PCA) behaviors provide a powerful and well-validated framework for understanding individual differences in addiction vulnerability. These behavioral phenotypes are remarkably stable across time and heritable across generations, and they robustly predict a range of addictive-relevant outcomes, including the rapid acquisition of drug-self administration, escalation of drug seeking, resistance to extinction, and heightened cue-induced reinstatement after abstinence (Morrow et al. 2011; Saunders and Robinson 2013; Tomie et al. 2008; Ahrens et al., 2016). ST reflects the attribution of salience to reward-predictive cues, a motivational process that parallels core features of human addiction such as elevated cue-reactivity, attentional capture by drug-associated stimuli, cue-evoked craving, and relapse triggered by environmental reminders of drug use. Conversely, GT is associated with greater flexibility and reduced cue-sensitivity, aligning with lower relapse risk. Thus, individual variation in the ST/GT behavior models trait-like, cross-species differences in cue-motivated responding making this paradigm a strong translational tool for studying addiction liability.

This translational relevance underscores the value of the ST/GT framework dissecting the neurobiological mechanisms that confer vulnerability to substance use disorders. While dopamine has long been recognized as central to incentive salience attribution, growing evidence demonstrates that cholinergic, serotonergic, adrenergic, opioid and adenosine signalling also shape these behavioral tendencies. Identifying how these neuromodulators bias individuals toward ST or GT has important clinical implications: it can clarify which neurotransmitter systems mediate susceptibility to addiction, and it may illuminate why certain individuals are more prone to relapse when exposed to drug-associated cues. Relapse, a defining feature of addiction, can be triggered by environmental stimuli months or even years after abstinence (Frawley and Smith 1992; Lee et al. 2022; Moos and Moos 2006; Zhu et al. 2018). Understanding the mechanisms that drive heightened cue-reactivity in STs may therefore reveal predictive markers of addiction risk that emerge well before drug exposure and identify novel targets for relapse prevention.

### The use of the Pavlovian conditioned approach index (PCA)

To contextualize the neurobiological differences between STs and GTs, it is first necessary to discuss the Pavlovian Conditioned Approach Index (PCA) for several reasons, a widely used measure for quantifying individual variation in cue-directed behavior (Meyer et al. 2012). This index is a composite score that considers multiple dependent variables. It is typically calculated using three behavioral measures during the cue (conditioned stimulus) period:

**Probability difference score**

$$P=\left(Probability\;of\;lever\;contact\right)-\left(Probability\;of\;food\;cup\;entry\right)$$

**Response bias score**

$$R=\frac{Number\;of\;lever\;contacts-Number\;of\;food\;cup\;entries}{Number\;of\;lever\;contacts+Number\;of\;food\;cup\;entries}$$

**Latency difference score**

$$L=\frac{Latency\;to\;food\;cup-Latency\;to\;lever\;contact}{Cue\;duration}$$

The PCA Index is computed as the average of these three normalized scores (PCA Index = *P + R + L* */3).* Each score ranges from 1 to + 1. Positive values (+ 0.5 to + 1.0) indicate ST bias. Negative values (−0.5 to −1.0) indicate GT bias. Intermediate values (–0.5 to + 0.5) indicate IT phenotype. It’s worth noting that some studies don’t generate PCA scores, but we’ve still included them in this review (Angelyn et al. 2021; Palmatier et al. 2013; Stringfield et al. 2017, 2018; Winstanley et al. 2004).

### Neurobiology of sign and goal tracking

The dopamine system is one of the most extensively studied neurotransmitter systems in relation to ST and GT (Gyawali et al. 2023; Iglesias et al. 2023; Lopez et al. 2015; Roughley and Killcross 2019). Multiple laboratories using pharmacological (Bacharach et al. 2023), optogenetic (Iglesias et al. 2023), and electrochemical approaches (Flagel et al. 2011) have consistently linked increased dopamine in the nucleus accumbens (NAc) to ST, particularly when the cue is present (Gillis and Morrison 2019). Lesioning the NAc core region has been shown to result in deficits in ST behavior (Cardinal et al. 2002; Chang et al. 2012). Likewise, depleting dopamine (Parkinson et al. 2002) or blocking dopamine receptors (Saunders and Robinson 2012) has a similar result. While both STs and GTs exhibit phasic dopamine release in the NAc in response to reward-predictive cues, only STs’ cue-evoked responses increase while reward-evoked responses diminish across training. This shift, absent in GTs, suggests the acquisition of ST uniquely relies on mesolimbic dopamine-encoded reward prediction errors (Flagel et al. 2011). While the role of dopamine is important, its role has been comprehensively reviewed elsewhere (Tomie et al. 2008) and will not be the focus of this article. Readers are referred to Tomie et al. (2008) for a detailed discussion of dopaminergic contribution to ST and GT phenotypes. Here, we aim to survey the literature in an assessment of other neurotransmitter systems that may also play a role in PCA and ST/GT behavior.

### The role of the endocannabinoid system in sign and goal tracking

Given the well-established role that cue-evoked dopamine release in the NAc plays in ST behavior, the endocannabinoid system is a strong candidate neuromodulatory system underlying individual differences in cue-motivated responding. Endocannabinoid signalling in the ventral tegmental area (VTA) exerts powerful control over mesolimbic dopamine release transmission (Cheer et al. 2000, 2004, 2007; Lupica and Riegel 2005; Lupica et al. 2004; Oleson and Cheer 2012; Wenzel et al. 2018), and multiple studies demonstrate that endocannabinoids directly modulate the motivational value of reward-predictive cues. For example, pharmacological blockade of endocannabinoid CB1 receptors in the VTA reduces lever contacts and lever approach probability in STs and suppresses cue-evoked NAc dopamine release (Bacharach et al. 2018, 2023; Oleson and Cheer 2012). Similarly, administration the non-selective cannabinoid agonist CP-55,940 during PCA training in rats produced a dose-dependent decrease in ST, reflected in fewer lever contacts and lower PCA indices, without affecting GT behavior (Gheidi et al. 2020).

Critically, Kruse et al. (2019) provide compelling evidence that the endocannabinoid system can shape long-term vulnerability to ST phenotypes. Using a model of adolescent voluntary THC ([−]-trans-Δ^9^-tetrahydrocannabinol) consumption, the authors showed that adolescent males, but not females, displayed enhanced ST in adulthood, along with selective downregulation of CB1 receptor-expressing glutamatergic terminals in the VTA. These long-lasting, sex-specific alterations in CB1-mediated control of VTA excitatory inputs suggest that developmental perturbations of endocannabinoid signaling shift dopamine-dependent cue-learning mechanisms toward a heightened incentive-salience profile. Kruse et al. (2019) thus directly link adolescent cannabinoid exposure with an adult ST bias via persistent endocannabinoid-driven changes in the VTA circuitry, supporting the model that endocannabinoid function contributes causally to individual variation in cue-motivated behavior.

Radioligand autoradiography and site-specific manipulations (e.g., fatty acid amide hydrolase (FAAH), the enzyme that degrades endogenous cannabinoids) further converge on the VTA as a key locus where CB1 signalling shapes ST behavior. STs exhibited elevated CB1 expression in the infralimbic cortex compared to GTs (Gheidi et al. 2020) and intra-VTA CB1 antagonism selectively reduces lever-directed responding in STs (Bacharach et al. 2023). Fiber photometry and optogenetic work confirm that CB1 receptors modulate VTA- NAc dopamine dynamics in a manner than tracks with cue-evoked approach, indicating that endocannabinoid activity gates the expression of incentive salience. Inhibition of VTA neurons with halorhodopsin while recording in the NAc reduced lever contacts selectively in STs. Similarly, intra-VTA CB1 receptor blockade with rimonabant decreased ST in STs but not intermediates (Bacharach et al. 2023).

Taken together, these findings indicate that endocannabinoids, particularly CB1 receptor signaling in the VTA◊ NAc circuit (Bacharach et al. 2023) and infralimbic cortex (Gheidi et al. 2020), plays a selective and mechanistically coherent role in modulating mesolimbic dopamine transmission that promotes ST behavior. Across studies, manipulating CB1 activity, whether by systemic antagonism, site-specific VTA blockade, or cannabinoid agonist administration, consistently alters cue-driven responding in STs, but not GTs, suggesting that the endocannabinoid system specifically modulates the attribution of incentive salience rather than general reward learning or goal-directed processes. Moreover, adolescence is a critical period for endocannabinoid-dopamine circuit maturation (Meyer et al. 2018; Tseng and Molla 2025). Given that ST and cue-driven drug seeking rely on intact CB1 receptor-mediated facilitation of VTA dopamine release, adolescent cannabis exposure, which directly targets CB1 receptors, could bias individuals toward altered emotional or cue salience processing, potentially enhancing vulnerability to drug-related cues or impairing adaptive control over reward seeking (Draycott et al. 2014; Ferland et al. 2023; Tseng and Molla 2025). Developmental perturbations (Kruse et al. 2019), systemic pharmacology, and region-specific CB1 manipulations converge on the conclusion that endocannabinoid tone biases individuals towards or away from high cue-reactivity phenotypes. This identifies CB1-modulated VTA dopamine plasticity as a critical pathway through which endocannabinoid signalling contributes to addiction-relevant susceptibility to cue-driven drug-seeking.

### The role of the cholinergic system in sign and goal tracking behavior

Nicotine has long been recognized for its reinforcement-enhancing properties, particularly its ability to increase the motivational value of reward-predictive cues. Several studies have demonstrated that nicotine reliably increases responding for otherwise weak or neutral stimuli, enhancing both primary reinforcement and the incentive value of associated cues (Donny et al. 2003; Chaudhri et al. 2007; Olausson et al. 2004). Moreover, researchers have also shown that nicotine functions not only as a primary reinforcer, but also as a potent conditioned reinforcer. More specifically, nicotine-paired cues acquire heightened incentive salience, drive approach behavior, and promote cue-controlled responding (Palmatier et al. 2007; Murray and Bevins 2007). Collectively, this literature demonstrates that activation of nicotinic acetylcholine receptors (nAChRs) amplifies the motivational significance of reward-predictive cues, directly aligning nicotine’s behavioral profile with incentive-salience processes central to ST.

More broadly, the cholinergic system influences attentional control and reward processing through widespread projections from the basal forebrain to cortical and limbic regions (Ballinger et al. 2016). Acetylcholine acts at two main receptor classes: fast-acting nAChRs, ligand-gated ion channels that modulate excitability and neurotransmitter release, and slower muscarinic acetylcholine receptors (mAChRs), G-protein coupled receptors, which regulate longer-timescale signalling (Akaike and Izumi 2018). Nicotinic receptors in the VTA can directly modulate dopamine neuron activity (Liu et al. 2012), linking cholinergic signaling to the attribution of incentive salience, suggesting a mechanistic route through cholinergic tone may bias individuals toward either ST or GT phenotypes. Consistent with this framework, manipulations of the cholinergic system can shift PCA behavior. In the context of developing non-invasive pharmacological approaches for substance use disorders, we examined the effects of muscarinic (Scopolamine) and nicotinic (Mecamylamine) receptor antagonists on PCA behavior. Our results showed that pretreatment with scopolamine dose-dependently decreased ST (reducing lever contacts, lever approach probability, and PCA index) and increased GT, whereas pretreatment with mecamylamine selectively reduced lever-directed responding without significantly changing magazine entries or reclassifying animals into different phenotypic categories (Gheidi et al. 2023). These findings reinforce the view that cholinergic signaling is deeply embedded in the neurobiology of cue-driven approach behavior and may contribute to individual differences in addiction-relevant cue responsivity.

Systemic nicotine administration has produced mixed effects on PCA behavior. In some studies, nicotine biased CPA measures (approach, probability, and latency) toward GT (increasing magazine entries and food cup approach probability) without altering ST parameters (Angelyn et al. 2021), while in others, acute nicotine administration increased ST behavior, even in nicotine-naïve rats, suggesting that nicotine can enhance the incentive salience of cues, independent of reinforcement history (Overby et al. 2018). Additional reports have indicated that nicotine increases both ST and GT (Palmatier et al. 2013; Stringfield et al. 2017), with sex differences in ST reported (Stringfield et al. 2019). Moreover, the tendency to ST predicts cue-induced reinstatement during nicotine self-administration, and is enhanced by nicotine, but not alcohol (Versaggi et al. 2016).

One study also showed that independent of nicotine exposure, drug-naïve female rats displayed increased cue-directed behavior compared to males and increased ST citing that brain-derived neurotrophic factor did not play a role (Stringfield et al. 2019). Females will take more nicotine in extended-access models (Chellian et al. 2024). Female rodents have a greater proportion of dopamine projecting neurons compared to males (Kritzer and Creutz 2008), and VTA-dopamine neurons are sensitive to circulating levels of estradiol (E2) (Kokane and Perrotti 2020). Sex differences in nicotine receptor density also exist. For instance, in female rodents, nicotine does not lead to an upregulation of β2 nAChRs, whereas it does in males (Koylu et al. 1997). Evidence in humans indicates that men tend to smoke for nicotine’s reinforcing effects, while women are more influenced by non-nicotine factors such as negative affective regulation (related to stress), or conditioning (likely due to the fact that they show less nicotine-evoked dopamine release in the ventral striatum) (Verplaetse et al. 2025). Women also face greater difficulty quitting and respond less to nicotine replacement therapies (Perkins et al. 1999; Verplaetse et al. 2025), highlighting the need to understand sex-specific neurochemical mechanisms of nicotine use. Nicotine occupies a distinctive position among drugs of abuse. For individuals without prior exposure, it often produces strong aversive effects (Fowler and Kenny 2014), and its modest euphoric properties are subject to rapid tolerance (Benowitz 2010). Yet, despite these features and well-known negative health consequences, nicotine remains highly addictive, with cessation proving difficult for many users. One mechanism that helps reconcile this paradox is nicotine’s ability to enhance reinforcement: as a reinforcement enhancer, it can amplify the incentive value of other stimuli, including cues associated with alcohol and other drugs of abuse Perkins et al. 2017).

Interestingly, Pitchers et al. (2017) examined how a discriminative stimulus (a cue signaling cocaine availability) influenced relapse in ST and GT rats, and looked at the role of basal forebrain cholinergic activity in this process. The discriminative stimulus triggered stronger reinstatement in GTs compared to STs, and cholinergic disruption reduced cue-induced cocaine seeking in GTs. In STs, reinstatement was not affected, indicating distinct neural mechanisms underlying relapse in the two phenotypes. Cholinergic neurons likely influence relapse by modulating attentional and cognitive control processes, making GTs particularly sensitive to discriminative stimuli. Nicotine exposure has also been shown to promote behavioral inflexibility (Stringfield et al. 2018): nicotine-treated rats persisted in responding to outdated cue locations during reward-omission trials and in cue–reward reassignment tasks, suggesting impaired reward updating. One study examining the how acetylcholine receptors in the NAc core influenced the flexibility of cue-motivated behavior described nAChRs and mAChRs as having opposing roles in regulating the persistence and restructuring of cue-directed motivation (Townsend et al. 2023). Using an omission procedure (where lever deflection during cue presentation cancelled reward delivery), the authors tested how blocking nAChRs or mAChRs receptors affected behavioral adaptation. They found that nAChRs blockade promoted flexibility later but initially heightened cue engagement, whereas mAChR blockade showed more flexibility from the outset. Thus, modulating accumbal acetylcholine signaling may offer a means to reshape maladaptive cue-driven behaviors, providing a potential therapeutic avenue for conditions characterized by rigid, compulsive responses to reward-associated cues.

Mechanistic studies have probed the cholinergic contributions to attentional processes in STs and GTs. Using chemogenetic hM4Di (human M4 muscarinic inhibitory) DREADDs (Designer Receptors Exclusively Activated by Designer Drugs) inhibition of cholinergic neurons in the horizontal limb of the diagonal band (HBD), it was found that suppression of these neurons impaired sustained attention task performance in GTs but not STs, reducing goal-hit rates in the former (Kucinski et al. 2022). These findings indicate that superior attentional performance in GTs depends on intact cholinergic function in the basal forebrain. Cortical acetylcholine availability also appears to shape tracking phenotype. Pharmacological inhibition of presynaptic choline transporters (CHT) with VU6001221 in GT rats induced a phenotypic shift towards ST. Reflected in increased lever contacts, decreased lever latency, reduced magazine entries, and increased magazine entry latency. Furthermore, basal forebrain electrostimulation (BF-ES) was performed in rats anesthetized with urethane and ultimately enhanced CHT expression in GT, but not ST rats, thus indicating that GT rats possess a greater capacity to mobilize CHT to maintain GT behavior during attentional demand (Koshy Cherian et al. 2017).

In sum, cholinergic signaling influences ST and GT in a receptor-specific and context-dependent manner. Muscarinic antagonism reduces ST and promotes GT, while nicotinic antagonism selectively suppresses ST, without affecting GT. Nicotine itself can bias behavior toward either phenotype depending on dose, sex, and task parameters, consistent with its reinforcement-enhancing effects and complex modulation of dopamine signaling. Mechanistically, basal forebrain cholinergic function and cortical acetylcholine availability appear critical for sustaining GT behavior, whereas disruptions in these systems, whether via pharmacological blockade, attentional impairment, or altered receptor regulation, tend to bias animals toward ST.

### The role of the serotonergic system in sign and goal tracking

Compared to other neurotransmitter systems, relatively few studies have examined serotonin’s role in ST & GT behavior. Early work, primarily in the context of impulsivity, showed that intracerebroventricular infusions of the 5-HT neurotoxin 5,7-dihydroxytryptamine (5,7-DHT). dihydroxytryptamine increased approaches to both CS- and CS + with lesioned animals making more approaches to the CS + during omission trials (Winstanley et al. 2004) suggesting impaired discrimination and inhibitory control. Similarly, food-restricted serotonin knockout rats also did not differ from wildtype controls in either ST or GT responses (Nonkes et al., 2013) indicating that global serotoninergic disruptions do not uniformly shift PCA phenotypes.

More recent pharmacological studies provide clearer evidence of serotonergic modulation. Systemic selective serotonin reuptake inhibitors (SSRIs) administration reliably decreases ST. Escitalopram produced a dose- dependent decrease in ST, reducing lever contacts and lever-approach probability and increasing latency to approach the lever, but only in animals that exhibit a ST phenotype, without comparable effects in GTs (Holden 2023). Likewise, citalopram, escitalopram, and fluoxetine reduced lever-directed behavior in male Sprague-Dawley rats, although effects on GT varied across compounds. These findings suggest that increasing extracellular serotonin attenuate the motivational pull of conditioned cues, raising the possibility that SSRIs may reduce cue-triggered relapse by dampening ST tendencies (Holden 2023).

Campus et al. (2016) provide the most detailed developmental analysis of serotonergic contributions to PCA. In this work, DBA/2J and C57BL/6J mice displayed robust ST during adolescence.However, only DBA/2J mice maintained a relaible ST phenotype into adulthood. Overall, PCA scores in mice were generally positive, consistent with a bias toward ST behavior. To test whether serotonergic signaling in the medial prefrontal cortex (mPFC) regulates this developmental trajectory, Campus et al. (2016) selectively depleted mPFC serotonin in adolescent C57BL/6J mice using the neurotoxin 5,7-dihydroxytryptamine (5,7-DHT). This manipulation caused C57BL/6J mice, to develop persistent adult ST behavior, mimicking the DBA/2J phenotype. These findings demonstrate that serotonergic tone in the mPFC during adolescence plays a critical role in constraining the emergence and persistence of ST behavior.

Taken together, the limited literature indicates that serotonin modulates ST and GT through multiple routes: (1) serotonergic disruption can impair cue discrimination and inhibitory control, increasing maladaptive cue engagement; (2) SSRIs reduce ST, suggesting that elevated serotonin dampens incentive-salience attribution; and (3) developmental serotonin depletion in the mPFC promotes persistent adult ST. Although sparse, these converging findings support serotonin as a key regulator of the balance between ST and GT, with alterations in serotonergic signaling, whether pharmacological or neurodevelopmental, biasing individuals toward or away from cue-directed behavior.

### The role of the adrenergic system in sign and goal tracking

Compared to other neuromodulators, the adrenergic system has received even less attention than the serotonergic system in the context of ST and GT. However, two recent studies provide important insight. Holden (2022, 2023) tested atomoxetine (a norepinephrine reuptake inhibitor) and guanfacine an alpha-2-adrenergic agonist) in rats performing the PCA task. Atomoxetine produced a dose-dependent suppression of both ST and GT, but the pattern differed by behavioral phenotype. ST was robustly reduced at all doses. Importantly, atomoxetine primarily reduced the intensity of lever and magazine-directed responding (PCA component scores) rather than fully converting ST animals into GT; many rats instead shifted toward intermediate phenotype scores. This included fewer lever contacts, shorter lever contact durations, reduced probability of approaching the lever during the conditioned stimulus, and increased latency to first elver contact. GT was only reduced at the highest dose tested. This included reduced magazine entries, lower probability of approaching the food cup, and increases latency to approach the magazine. This suggests that modest elevations in prefrontal norepinephrine preferentially disrupts cue-directed incentive behaviors (ST), whereas GT, which relies more on predictive value than incentive salience, only becomes disrupted when NE levels are sufficiently high to interfere with goal-directed approach or attentional selection. In contrast, guanfacine failed to alter either ST or GT across a wide dose range, indicating that not all adrenergic manipulations influence conditioned approach in the same way.

The divergent patterns map onto their pharmacological profiles. Both drugs result in increased extracellular norepinephrine (in the PFC specifically) and are used clinically to treat attention deficit and hyperactivity disorder (ADHD) (Arnsten and Pliszka 2011), yet their divergent outcomes highlight a critical point: the behavioral effects of norepinephrine depend on receptor subtype engagement (α1, α2, β) and circuit-level action. Atomoxetine increases extracellular norepinephrine broadly by blocking the norepinephrine transporter, thereby engaging multiple adrenergic receptor subtypes (Garnock-Jones and Keating 2009) across cortical and subcortical circuits. Guanfacine, by contrast, selectively stimulates presynaptic α2A autoreceptors, which reduces norepinephrine release through negative feedback in some pathways (Arnsten 2020). Atomoxetine enhances norepinephrine signaling across multiple receptor systems, while guanfacine dampens norepinephrine output in certain pathways. In ST and GT, the outcome seems to depend not only on the amount of norepinephrine but on where and how it is acting. Thus, despite both raising extracellular norepinephrine in the PFC, their net circuit effects differ dramatically, highlighting that adrenergic modulation of ST and GT depends on receptor specificity, circuit engagement, and the degree of noradrenergic elevation. Accordingly, the adrenergic system’s role in cue-motivated behavior is mechanism-dependent because the same neurotransmitter produces opposite behavioral outcomes depending on dose, receptor subtype, and circuit-level action.

The timing of adrenergic modulation is equally critical. Pasquariello et al. (2018) demonstrated that reducing norepinephrine synthesis with the dopamine β-hydroxylase inhibitor disulfiram suppressed ST only when administered before Pavlovian training, with no impact when given after ST was already acquired. Blockade of α1-adrenergic receptors (prazosin) or β-adrenergic receptors (propranolol) also selectively reduced ST without affecting GT. Together, these studies show that ST acquisition and expression depend on intact norepinephrine transmission, whereas GT is comparatively insensitive to adrenergic manipulation, again supporting a more norepinephrine-dependent motivational mechanism in ST.

Within a broader framework, these pharmacological effects align with the known role of the locus coeruleus (LC), the brain’s primary source of norepinephrine (Dahlström and Fuxe 1965). LC neurons exhibit phasic firing to predictive and salient cues (Aston-Jones et al. 1997) and support attentional orienting/shifts, behavioral flexibility, and adaptive cue-outcome updating (when an organism changes what it expects a cue to predict) (Aston-Jones et al. 2000; Grella and Donaldson 2024; McBurney-Lin et al. 2022). Importantly, LC norepinephrine activity increases when contingencies change, facilitating updating during extinction, reversal learning, and prediction-error conditions (Grella et al. 2021; Janitzky et al. 2015; Rorabaugh et al. 2017). Here, phasic signaling specifically refers to rapid, event-locked bursts of LC activity that enhance learning about conditioned stimuli. This helps adjust cue-driven behavior when the cue’s meaning shifts. In contrast, dysregulated or exaggerated adrenergic signaling may exaggerate the motivational value of reward-predictive cues, contributing to the persistence of ST even when contingencies change.

Overall, the literature indicates that ST, but not GT, is strongly dependent on adrenergic signaling, and that the behavioral consequences of adrenergic manipulation depend on when norepinephrine is altered (during learning vs. after), where in the circuit it is modulated (PFC vs. subcortical targets), and which receptors are engaged. This unified framework clarifies why adrenergic drugs produce heterogeneous effects across studies and underscores norepinephrine’s selective contribution to the motivational component of cue-directed behavior.

### The role of the opioid system in sign and goal tracking

Given that ST reflects the attribution of incentive salience to reward-predictive cues, whereas GT reflects the reward itself, the opioid system may regulate both behaviors by modulating motivational value. One study examined whether direct stimulation of µ-opioid receptors in the central amygdala (CeA) could influence ST & GT in rats. Site-specific microinjections of DAMGO, a selective µ-opioid receptor agonist (D-Ala2,NMePhe4,Gly-ol5enkephalin) enhanced approach behaviors toward each phenotype’s preferred target. In GTs, DAMGO increased contacts with the food magazine, while in STs, it increased lever interactions (DiFeliceantonio and Berridge 2012). These effects indicate that CeA µ-opioid activation amplifies the motivational salience of reward-related cues and outcomes, intensifying existing behavioral biases rather than shifting animals between phenotypes. In the same study, DAMGO increased lever presses and food cup entries only in response to reward-predictive cues, with no effect on cues unassociated with reward. This demonstrates that the observed increases were linked to reward expectation, rather than general hyperactivity.

In addition to µ-opioid receptors, the brain also expresses δ-and κ-opioid receptors (KORs) each with distinct functional profiles and anatomical distributions (Erbs et al. 2015; Le Merrer et al. 2009). Although direct evidence linking these receptor systems to ST & GT is limited, research in reward-related circuits points to their potential roles. δ-opioid agonists like DPDPE (D-Pen 2, D-Pen 5] enkephalin) and deltorphin II, similar to DAMGO and morphine, induce neuronal hyperpolarization by activating inhibitory Gi/o G-proteins, reducing the influx of calcium ions and opening G-protein-dependent inward rectifier potassium channels (GIRKS) (Reeves et al. 2022). This suggests that δ-opioid receptors may be able to modulate excitability in brain regions involved in reward processing and may potentially act in similar ways to µ-opioid modulation. In fact, stimulating opioid receptors within the NAc shell has been shown to enhance hedonic “liking” responses (Castro & Berridge, 2014). This highlights the potential for these receptors to influence incentive salience and cue attraction.

In the above study, they found that the κ-opioid receptor agonist, U50488H, enhanced hedonic “liking” responses (Castro & Berridge, 2014). KOR activation produces a well-established aversive state, largely via dynorphin-mediated suppression of mesolimbic dopamine (Bals-Kubik et al. 1993; Grella et al. 2014; Land et al. 2009); thus, its role in reward processing is not well-understood. With respect to conditioned place preference, low doses of the κ-opioid receptor agonist salvinorin A induced preference but high doses induced aversion (Braida et al. 2008). Similarly, rats will self-administer low doses but not high doses (Braida et al. 2008). These findings raise the possibility that mild κ-opioid receptor activation could, like µ-opioid stimulation, enhance the motivational salience of cues or outcomes thereby amplifying pre-existing ST or GT tendencies. In contrast, high dose κ-opioid receptor activation would potentially suppress ST (especially dopamine-dependent effects) consistent with the dopaminergic suppression (Gray et al. 1999) and dysphoric states associated with robust κ-opioid receptor stimulation (Carlezon et al. 2006). Moreover, this aversion competes with appetitive motivation and can reduce cue attraction independently of incentive-salience mechanisms. In the context of ST/GT, such aversive motivational signaling would be expected to blunt ST, which depends on dopamine-mediated incentive salience, while more moderately affecting GT behaviors that rely on cognitive expectancy. Importantly, the aversive component of KOR activation may mask or override typical cue-driven approach responses. Because ST is particularly sensitive to dopamine suppression and negative affect, KOR-induced aversion could selectively diminish CS-directed approach, although this has yet to be empirically tested in ST/GT assays.

### The role of the adenosine system in sign and goal tracking

As a psychostimulant, caffeine modulates reinforcement sensitivity primarily through adenosine receptor antagonism, leading to downstream increases in dopaminergic neurotransmission (Hsu et al. 2010). These effects have promoted investigation into caffeine’s influence on ST and GT behavior given its potential to enhance motivational salience and cognitive performance. In one study, rats received caffeine or saline prior to each session of a PCA procedure in which a lever predicted food delivery (Holden et al. 2022). ST and GT were quantified via lever contacts, food magazine entries, and associated latencies and probabilities. Caffeine administration produced a significant, dose-dependent increase in ST (lever contacts, lever-approach probability, and PCA index) behavior without affecting GT. This pattern indicates that caffeine selectively enhances the motivational value attributed to reward-predictive cues, rather than the reward location itself. The authors suggest that this effect may be mediated by increased dopamine release in the NAc and elevated acetylcholine levels in the medial PFC.

Importantly, these effects are consistent with a broader literature on adenosine A₂A receptor antagonists, which can facilitate dopaminergic transmission in mesolimbic reward pathways (specifically at D2 receptors), thereby enhancing the incentive salience of conditioned cues (Prasad et al. 2021; Prediger et al. 2005). Together, these results suggest that caffeine may enhance sensitivity to conditioned cues without altering general reward approach, raising the possibility that common stimulants could exacerbate cue-triggered relapse risk in individuals predisposed to ST.

## Discussion

The studies reviewed here demonstrate that ST and GT behavior in rodents are shaped by multiple neurotransmitter systems, each contributing distinct but highly interconnected pathways that converge on dopaminergic function. Although dopamine remains central to attributing incentive salience, its activity is modulated by cholinergic, serotonergic, adrenergic, opioid, and adenosine systems in ways that differentially bias cue-motivated behavior. Cholinergic manipulations often bias GTs toward ST, likely by disrupting prefrontal and striatal cholinergic inputs that normally regulate attention and thereby constrain dopamine-driven cue attraction. Serotonin generally reduces ST, and several studies suggest this occurs through serotonergic inhibition of mesolimbic dopamine neuron firing, though effects vary by strain and drug-specific pharmacology. Adrenergic manipulations primarily reduce ST while generally not affecting GT except at higher doses, consistent with noradrenergic modulation of dopamine release in the NAc and its role in amplifying cue-triggered motivation. This indicates a dependence on noradrenergic signaling for cue-directed behavior. Opioid receptor stimulation amplifies pre-existing phenotype tendencies reflecting µ- and κ-opioid modulation of dopamine tone that enhances incentive salience without necessarily shifting animals between ST and GT categories. Finally, nicotine and caffeine increase ST behavior in a mechanistic and dose-dependent manner, which aligns with their well-established capacity to potentiate dopaminergic responses to reward-predictive cues. This also underscores the potential for common stimulants to heighten vulnerability to cue-driven relapse. Notably, while some sex-differences have been reported, the majority of these studies were done solely in males, and thus the neuromodulatory mechanisms described here may not generalize to females, and sex-specific contributions of these neuromodulators may differ in important ways.

Across studies, these neuromodulators influence ST and GT behavior through partially distinct but highly interacting circuit nodes, particularly the NAc, PFC, and LC. Dopamine-dependent incentive salience attribution is centered in the NAc core (Cardinal et al. 2002; Chang et al. 2012; Gillis and Morrison 2019; Bacharach et al. 2023; Iglesias et al. 2023; Flagel et al. 2011; Saunders and Robinson 2012; Tomie et al. 2008), where cholinergic (Townsend et al. 2023), endocannabinoid (Bacharach et al. 2018, 2023; Oleson and Cheer 2012), adenosingergic (Hsu et al. 2010; Prasad et al. 2021; Prediger et al. 2005) and opioid (Castro & Berridge, 2014) signaling modulate dopamine release dynamics to bias cue-directed behavior. Serotonergic and adrenergic systems additionally shape prefrontal functions, including attentional control and behavioral flexibility (Arnsten 2020; Arnsten and Pliszka 2011; Puig and Gulledge 2011). Additionally, the LC provides noradrenergic input to both the PFC and NAc, regulating arousal-based gain and amplifying the motivational significance of cues (Aston-Jones et al. 1997). Thus, the effects of these neurotransmitter system manipulations on ST/GT phenotypes are best understood not in isolation, but as neuromodulatory influences on a broader LC–PFC–NAc circuit that determines cue reactivity, behavioral regulation, and susceptibility to ST behavior.

A clearer understanding of how these neuromodulatory systems bias individuals toward ST or GT also highlights several potential pharmacological targets relevant for reducing cue-driven relapse vulnerability. Cholinergic modulation of attentional control and top-down suppression suggests that α7 nicotinic and M1 muscarinic receptors may serve as targets for dampening maladaptive cue attraction (Gheidi et al. 2023). Serotonergic influences on behavioral inhibition point to 5-HT2A-C, 5-HT1B receptors, already implicated in impulsivity and drug seeking, as promising mechanisms for reducing ST tendencies (Bevilacqua et al., 2010; Higgins et al., 2012; Anastasio et al. 2011; Nautiyal et al. 2015). Noradrenergic regulation of cue-evoked arousal through α2A and β-adrenergic receptors may offer opportunities to modulate excessive cue reactivity and enhance cognitive control over drug-associated stimuli (Arnsten 2020). Likewise, opioid receptor manipulations, particularly κ-opioid receptor antagonists, which reduce dysphoric and stress-enhanced cue responsivity, could mitigate vulnerabilities in individuals who strongly attribute incentive salience to cues (Gray et al. 1999; Carlezon et al. 2006). Finally, adenosinergic modulation of dopamine signaling through A1 and A2A receptors provides another route for altering motivational value assignment without directly targeting dopamine transmission (Quarta et al. 2004). Together, these neuromodulatory pathways expand the set of therapeutic leverage points beyond dopamine alone and underscore the practical value of ST/GT research for identifying treatments aimed at reducing cue-triggered relapse risk in substance use disorders.

Collectively, these findings reinforce that dopamine is not an isolated driver of ST and GT but instead, sits at the center of a broader neuromodulatory network. Disentangling how each system shapes dopaminergic firing, release, and receptor activation offers a more mechanistic understanding of why individuals differ in their susceptibility to cue-driven relapse. Future research should therefore move beyond single-system manipulations and examine how these neuromodulators interact under conditions of stress, genetic variation, and chronic drug exposure. Such work will be essential for developing individualized interventions that target system-level dysfunction rather than dopamine alone.

## Data Availability

The raw data is not available and was not used.

## References

[CR1] Ahrens AM, Singer BF, Fitzpatrick CJ, Morrow JD, Robinson TE (2016) Rats that sign-track are resistant to Pavlovian but not instrumental extinction. Behav Brain Res 296:418–430. 10.1016/j.bbr.2015.07.055 PMC4681397 26235331

[CR2] Akaike A, Izumi Y (2018) Nicotinic Acetylcholine Receptor Signaling in Neuroprotection [Internet]. In A. Akaike, S. Shimohama, & Y. Misu (Eds.). https://www.ncbi.nlm.nih.gov/books/NBK543551/?utm_source=chatgpt.com 31314229

[CR3] Anastasio NC, Stoffel EC, Fox RG, Bubar MJ, Rice KC, Moeller FG, Cunningham KA (2011) Serotonin (5-hydroxytryptamine) 5-HT(2A) receptor: association with inherent and cocaine-evoked behavioral disinhibition in rats. Behav Pharmacol 22(3):248–261. 10.1097/FBP.0b013e328345f90d PMC3124821 21499079

[CR4] Angelyn H, Loney GC, Meyer PJ (2021) Nicotine enhances goal-tracking in ethanol and food Pavlovian conditioned approach paradigms. Front Neurosci 15:561766. 10.3389/fnins.2021.561766 PMC8416423 34483813

[CR5] Arnsten AFT (2020) Guanfacine’s mechanism of action in treating prefrontal cortical disorders: successful translation across species. Neurobiol Learn Mem 176:107327. 10.1016/j.nlm.2020.107327 PMC7567669 33075480

[CR6] Arnsten AF, Pliszka SR (2011) Catecholamine influences on prefrontal cortical function: relevance to treatment of attention deficit/hyperactivity disorder and related disorders. Pharmacol Biochem Behav 99(2):211–216. 10.1016/j.pbb.2011.01.020 PMC3129015 21295057

[CR7] Aston-Jones G, Rajkowski J, Kubiak P (1997) Conditioned responses of monkey locus coeruleus neurons anticipate acquisition of discriminative behavior in a vigilance task. Neuroscience 80(3):697–715. 10.1016/s0306-4522(97)00060-2 9276487

[CR8] Aston-Jones G, Rajkowski J, Cohen J (2000) Locus coeruleus and regulation of behavioral flexibility and attention. Prog Brain Res 126:165–182. 10.1016/s0079-6123(00)26013-5 11105646

[CR9] Bacharach SZ, Nasser HM, Zlebnik NE, Dantrassy HM, Kochli DE, Gyawali U, Cheer JF, Calu DJ (2018) Cannabinoid receptor-1 signaling contributions to sign-tracking and conditioned reinforcement in rats. Psychopharmacology 235(10):3031–3043. 10.1007/s00213-018-4993-6 PMC6344029 30109373

[CR10] Bacharach SZ, Martin DA, Stapf CA, Sun F, Li Y, Cheer JF, Calu DJ (2023) Decreased ventral tegmental area CB1R signaling reduces sign-tracking and shifts cue-outcome dynamics in rat nucleus accumbens. J Neurosci. 10.1523/jneurosci.1486-22.2023 PMC10286939 37208179

[CR11] Ballinger EC, Ananth M, Talmage DA, Role LW (2016) Basal forebrain cholinergic circuits and signaling in cognition and cognitive decline. Neuron 91(6):1199–1218. 10.1016/j.neuron.2016.09.006 PMC5036520 27657448

[CR12] Bals-Kubik R, Ableitner A, Herz A, Shippenberg TS (1993) Neuroanatomical sites mediating the motivational effects of opioids as mapped by the conditioned place preference paradigm in rats. J Pharmacol Exp Ther 264(1):489–495 8093731

[CR13] Benowitz NL (2010) Nicotine addiction. N Engl J Med 362(24):2295–2303. 10.1056/NEJMra0809890 PMC2928221 20554984

[CR14] Bevilacqua L, Doly S, Kaprio J, Yuan Q, Tikkanen R, Paunio T, Zhou Z, Wedenoja J, Maroteaux L, Diaz S, Belmer A, Hodgkinson CA, Dell’osso L, Suvisaari J, Coccaro E, Rose RJ, Peltonen L, Virkkunen M, Goldman D (2010) A population-specific HTR2B stop codon predisposes to severe impulsivity. Nature 468(7327):23–6. 10.1038/nature09629 PMC3183507 21179162

[CR15] Boakes R (1977) Performance on learning to associate a stimulus with positive reinforcement. In: Davis H, Hurwitz H (eds) Operant-Pavlovian Interactions. Lawrence Erlebaum Associates, pp 67–97

[CR16] Braida D, Limonta V, Capurro V, Fadda P, Rubino T, Mascia P, Zani A, Gori E, Fratta W, Parolaro D, Sala M (2008) Involvement of kappa-opioid and endocannabinoid system on salvinorin A-induced reward. Biol Psychiatry 63(3):286–292. 10.1016/j.biopsych.2007.07.020 17920565

[CR17] Campus P, Accoto A, Maiolati M, Latagliata C, Orsini C (2016) Role of prefrontal 5-HT in the strain-dependent variation in sign-tracking behavior of C57BL/6 and DBA/2 mice. Psychopharmacology (Berl) 233(7):1157–1169. 10.1007/s00213-015-4192-7 26728892

[CR18] Cardinal RN, Parkinson JA, Hall J, Everitt BJ (2002) Emotion and motivation: the role of the amygdala, ventral striatum, and prefrontal cortex. Neurosci Biobehav Rev 26(3):321–352. 10.1016/s0149-7634(02)00007-6 12034134

[CR19] Carlezon WA Jr., Béguin C, DiNieri JA, Baumann MH, Richards MR, Todtenkopf MS, Rothman RB, Ma Z, Lee DY, Cohen BM (2006) Depressive-like effects of the kappa-opioid receptor agonist salvinorin A on behavior and neurochemistry in rats. J Pharmacol Exp Ther 316(1):440–447. 10.1124/jpet.105.092304 16223871

[CR20] Castro DC, Berridge KC (2014) Opioid hedonic hotspot in nucleus accumbens shell: mu, delta, and kappa maps for enhancement of sweetness liking and wanting. J Neurosci 34(12):4239–4250. 10.1523/jneurosci.4458-13.2014 PMC3960467 24647944

[CR21] Chang SE, Wheeler DS, Holland PC (2012) Roles of nucleus accumbens and basolateral amygdala in autoshaped lever pressing. Neurobiol Learn Mem 97(4):441–451. 10.1016/j.nlm.2012.03.008 PMC3358568 22469749

[CR22] Chaudhri N, Caggiula AR, Donny EC, Booth S, Gharib M, Craven L, Palmatier MI, Liu X, Sved AF (2007) Self-administered and noncontingent nicotine enhance reinforced operant responding in rats: impact of nicotine dose and reinforcement schedule. Psychopharmacology 190(3):353–362. 10.1007/s00213-006-0454-8 PMC2838240 16847680

[CR23] Cheer JF, Kendall DA, Marsden CA (2000) Cannabinoid receptors and reward in the rat: a conditioned place preference study. Psychopharmacology (Berl) 151(1):25–30. 10.1007/s002130000481 10958113

[CR24] Cheer JF, Wassum KM, Heien ML, Phillips PE, Wightman RM (2004) Cannabinoids enhance subsecond dopamine release in the nucleus accumbens of awake rats. J Neurosci 24(18):4393–4400. 10.1523/jneurosci.0529-04.2004 PMC6729440 15128853

[CR25] Cheer JF, Wassum KM, Sombers LA, Heien ML, Ariansen JL, Aragona BJ, Phillips PE, Wightman RM (2007) Phasic dopamine release evoked by abused substances requires cannabinoid receptor activation. J Neurosci 27(4):791–795. 10.1523/jneurosci.4152-06.2007 PMC6672925 17251418

[CR26] Chellian R, Behnood-Rod A, Bruijnzeel AW (2024) Sex differences in nicotine intake and relapse behavior in nicotine-dependent adult wistar rats. Front Pharmacol 15:1415219. 10.3389/fphar.2024.1415219 PMC11464435 39391691

[CR27] Dahlström A, Fuxe K (1965) Evidence for the existence of an outflow of noradrenaline nerve fibres in the ventral roots of the rat spinal cord. Experientia 21(7):409–410. 10.1007/bf02139774 5870780

[CR28] Deroche-Gamonet V, Belin D, Piazza PV (2004) Evidence for addiction-like behavior in the rat. Science 305(5686):1014–1017. 10.1126/science.1099020 15310906

[CR29] DiFeliceantonio AG, Berridge KC (2012) Which cue to ‘want’? Opioid stimulation of central amygdala makes goal-trackers show stronger goal-tracking, just as sign-trackers show stronger sign-tracking. Behav Brain Res 230(2):399–408. 10.1016/j.bbr.2012.02.032 PMC3322261 22391118

[CR30] Donny EC, Chaudhri N, Caggiula AR, Evans-Martin FF, Booth S, Gharib MA, Clements LA, Sved AF (2003) Operant responding for a visual reinforcer in rats is enhanced by noncontingent nicotine: implications for nicotine self-administration and reinforcement. Psychopharmacology 169(1):68–76. 10.1007/s00213-003-1473-3 12774186

[CR31] Draycott B, Loureiro M, Ahmad T, Tan H, Zunder J, Laviolette SR (2014) Cannabinoid transmission in the prefrontal cortex bi-phasically controls emotional memory formation via functional interactions with the ventral tegmental area. J Neurosci 34(39):13096–13109. 10.1523/jneurosci.1297-14.2014 PMC6608340 25253856

[CR32] Erbs E, Faget L, Scherrer G, Matifas A, Filliol D, Vonesch JL, Koch M, Kessler P, Hentsch D, Birling MC, Koutsourakis M, Vasseur L, Veinante P, Kieffer BL, Massotte D (2015) A mu-delta opioid receptor brain atlas reveals neuronal co-occurrence in subcortical networks. Brain Struct Funct 220(2):677–702. 10.1007/s00429-014-0717-9 PMC4341027 24623156

[CR33] Ferland JN, Ellis RJ, Betts G, Silveira MM, de Firmino JB, Winstanley CA, Hurd YL (2023) Long-term outcomes of adolescent THC exposure on translational cognitive measures in adulthood in an animal model and computational assessment of human data. JAMA Psychiat 80(1):66–76. 10.1001/jamapsychiatry.2022.3915 PMC9685552 36416863

[CR34] Flagel SB, Robinson TE, Clark JJ, Clinton SM, Watson SJ, Seeman P, Phillips PE, Akil H (2010) An animal model of genetic vulnerability to behavioral disinhibition and responsiveness to reward-related cues: implications for addiction. Neuropsychopharmacology 35(2):388–400. 10.1038/npp.2009.142 PMC2794950 19794408

[CR35] Flagel SB, Clark JJ, Robinson TE, Mayo L, Czuj A, Willuhn I, Akers CA, Clinton SM, Phillips PE, Akil H (2011) A selective role for dopamine in stimulus-reward learning. Nature 469(7328):53–57. 10.1038/nature09588 PMC3058375 21150898

[CR36] Fowler CD, Kenny PJ (2014) Nicotine aversion: neurobiological mechanisms and relevance to tobacco dependence vulnerability. Neuropharmacology 76(0 0):533–544. 10.1016/j.neuropharm.2013.09.008 PMC3858456 24055497

[CR37] Frawley PJ, Smith JW (1992) One-year follow-up after multimodal inpatient treatment for cocaine and methamphetamine dependencies. J Subst Abuse Treat 9(4):271–286. 10.1016/0740-5472(92)90020-o 1336066

[CR38] Garnock-Jones KP, Keating GM (2009) Atomoxetine: a review of its use in attention-deficit hyperactivity disorder in children and adolescents. Paediatr Drugs 11(3):203–226. 10.2165/00148581-200911030-00005 19445548

[CR39] George O, Koob GF (2017) Individual differences in the neuropsychopathology of addiction. Dialogues Clin Neurosci 19(3):217–229. 10.31887/DCNS.2017.19.3/gkoob PMC5741105 29302219

[CR40] Gheidi A, Cope LM, Fitzpatrick CJ, Froehlich BN, Atkinson R, Groves CK, Barcelo CN, Morrow JD (2020) Effects of the cannabinoid receptor agonist CP-55,940 on incentive salience attribution. Psychopharmacology 237(9):2767–2776. 10.1007/s00213-020-05571-3 PMC7502542 32494975

[CR41] Gheidi A, Fitzpatrick CJ, Gregory JD, Morrow JD (2023) Nicotinic and muscarinic acetylcholine receptor antagonism dose-dependently decreases sign- but not goal-tracking behavior in male rats. Psychopharmacology 240(4):871–880. 10.1007/s00213-023-06328-4 PMC10599605 36795109

[CR42] Gillis ZS, Morrison SE (2019) Sign tracking and goal tracking are characterized by distinct patterns of nucleus accumbens activity. eNeuro. 10.1523/eneuro.0414-18.2019 PMC6419996 30886890

[CR43] Gray AM, Rawls SM, Shippenberg TS, McGinty JF (1999) The kappa-opioid agonist, U-69593, decreases acute amphetamine-evoked behaviors and calcium-dependent dialysate levels of dopamine and glutamate in the ventral striatum. J Neurochem 73(3):1066–1074. 10.1046/j.1471-4159.1999.0731066.x 10461896

[CR44] Grella SL, Donaldson TN (2024) Contextual memory engrams, and the neuromodulatory influence of the locus coeruleus. Front Mol Neurosci 17:1342622. 10.3389/fnmol.2024.1342622 PMC10875109 38375501

[CR45] Grella SL, Funk D, Coen K, Li Z, Lê AD (2014) Role of the kappa-opioid receptor system in stress-induced reinstatement of nicotine seeking in rats. Behav Brain Res 265:188–197. 10.1016/j.bbr.2014.02.029 PMC4082245 24583188

[CR46] Grella SL, Gomes SM, Lackie RE, Renda B, Marrone DF (2021) Norepinephrine as a spatial memory reset signal. Behav Pharmacol 32(7):531–548. 10.1097/fbp.0000000000000648 34417358

[CR47] Gyawali U, Martin DA, Sun F, Li Y, Calu D (2023) Dopamine in the dorsal bed nucleus of stria terminalis signals Pavlovian sign-tracking and reward violations. Elife. 10.7554/eLife.81980 PMC10219648 37232554

[CR48] Hearst ES, Jenkins HM (1974) Sign-tracking: the stimulus-reinforcer relation and directed action. Austin, TX, Psychonomic Society

[CR49] Higgins GA, Silenieks LB, Rossmann A, Rizos Z, Noble K, Soko AD, Fletcher PJ (2012) The 5-HT2C receptor agonist Lorcaserin reduces nicotine self-administration, discrimination, and reinstatement: relationship to feeding behavior and impulse control. Neuropsychopharmacology 37(5):1177–1191. 10.1038/npp.2011.303 PMC3306879 22189292

[CR50] Holden JM (2022) Effects of two kinds of noradrenergic ADHD medicines on sign-tracking and goal-tracking in male rats. Exp Clin Psychopharmacol 30(6):760–773. 10.1037/pha0000538 34968108

[CR51] Holden JM (2023) Effects of three serotonin reuptake inhibitors on sign-tracking in male Sprague-Dawley rats. Physiol Behav 268:114233. 10.1016/j.physbeh.2023.114233 37172639

[CR52] Holden JM, Salem A, Ng ZT, Barun Shrestha R, Tibbetts M, Miller C, Carroll KL (2022) Acute caffeine enhances sign-tracking in male Sprague-Dawley rats. Behav Processes 203:104782. 10.1016/j.beproc.2022.104782 36402408

[CR53] Hsu CW, Wang CS, Chiu TH (2010) Caffeine and a selective adenosine A2A receptor antagonist induce sensitization and cross-sensitization behavior associated with increased striatal dopamine in mice. J Biomed Sci 17(1):4. 10.1186/1423-0127-17-4 PMC2843608 20074377

[CR54] Iglesias AG, Chiu AS, Wong J, Campus P, Li F, Liu ZN, Bhatti JK, Patel SA, Deisseroth K, Akil H, Burgess CR, Flagel SB (2023) Inhibition of dopamine neurons prevents incentive value encoding of a reward cue: with revelations from deep phenotyping. J Neurosci. 10.1523/jneurosci.0848-23.2023 PMC10621773 37709540

[CR55] Janitzky K, Lippert MT, Engelhorn A, Tegtmeier J, Goldschmidt J, Heinze HJ, Ohl FW (2015) Optogenetic silencing of locus coeruleus activity in mice impairs cognitive flexibility in an attentional set-shifting task. Front Behav Neurosci 9:286. 10.3389/fnbeh.2015.00286 PMC4631833 26582980

[CR56] Kokane SS, Perrotti LI (2020) Sex differences and the role of estradiol in mesolimbic reward circuits and vulnerability to cocaine and opiate addiction. Front Behav Neurosci 14:74. 10.3389/fnbeh.2020.00074 PMC7251038 32508605

[CR57] Koshy Cherian A, Kucinski A, Pitchers K, Yegla B, Parikh V, Kim Y, Valuskova P, Gurnani S, Lindsley CW, Blakely RD, Sarter M (2017) Unresponsive choline transporter as a trait neuromarker and a causal mediator of bottom-up attentional biases. J Neurosci 37(11):2947–2959. 10.1523/JNEUROSCI.3499-16.2017 PMC5354335 28193693

[CR58] Koylu E, Demirgören S, London ED, Pöğün S (1997) Sex difference in up-regulation of nicotinic acetylcholine receptors in rat brain. Life Sci 61(12):Pl185–190. 10.1016/s0024-3205(97)00665-6 9315514

[CR59] Kritzer MF, Creutz LM (2008) Region and sex differences in constituent dopamine neurons and immunoreactivity for intracellular Estrogen and androgen receptors in mesocortical projections in rats. J Neurosci 28(38):9525–9535. 10.1523/jneurosci.2637-08.2008 PMC2613180 18799684

[CR60] Kruse LC, Cao JK, Viray K, Stella N, Clark JJ (2019) Voluntary oral consumption of ∆9-tetrahydrocannabinol by adolescent rats impairs reward-predictive cue behaviors in adulthood. Neuropsychopharmacology 44(8):1406–1414. 10.1038/s41386-019-0387-7 PMC6785709 30965351

[CR61] Kucinski A, Avila C, Sarter M (2022) Basal forebrain chemogenetic inhibition converts the attentional control mode of goal-trackers to that of sign-trackers. eNeuro. 10.1523/ENEURO.0418-22.2022 PMC9794377 36635246

[CR62] Land BB, Bruchas MR, Schattauer S, Giardino WJ, Aita M, Messinger D, Hnasko TS, Palmiter RD, Chavkin C (2009) Activation of the kappa opioid receptor in the dorsal Raphe nucleus mediates the aversive effects of stress and reinstates drug seeking. Proc Natl Acad Sci U S A 106(45):19168–19173. 10.1073/pnas.0910705106 PMC2776420 19864633

[CR63] Le Merrer J, Becker JA, Befort K, Kieffer BL (2009) Reward processing by the opioid system in the brain. Physiol Rev 89(4):1379–1412. 10.1152/physrev.00005.2009 PMC4482114 19789384

[CR64] Lee SH, Yi YH, Lee YI, Lee HY, Lim K-M (2022) Factors associated with long-term smoking relapse in those who succeeded in smoking cessation using regional smoking cessation programs. Medicine 101(31):e29595. 10.1097/md.0000000000029595 PMC9351863 35945709

[CR65] Liu L, Zhao-Shea R, McIntosh JM, Gardner PD, Tapper AR (2012) Nicotine persistently activates ventral tegmental area dopaminergic neurons via nicotinic acetylcholine receptors containing α4 and α6 subunits. Mol Pharmacol 81(4):541–548. 10.1124/mol.111.076661 PMC3310415 22222765

[CR66] Lopez JC, Karlsson RM, O’Donnell P (2015) Dopamine D2 modulation of sign and goal tracking in rats. Neuropsychopharmacology 40(9):2096–2102. 10.1038/npp.2015.68 PMC4613614 25759299

[CR67] Lovic V, Saunders BT, Yager LM, Robinson TE (2011) Rats prone to attribute incentive salience to reward cues are also prone to impulsive action. Behav Brain Res 223(2):255–261. 10.1016/j.bbr.2011.04.006 PMC3119757 21507334

[CR68] Lupica CR, Riegel AC (2005) Endocannabinoid release from midbrain dopamine neurons: a potential substrate for cannabinoid receptor antagonist treatment of addiction. Neuropharmacology 48(8):1105–1116. 10.1016/j.neuropharm.2005.03.016 15878779

[CR69] Lupica CR, Riegel AC, Hoffman AF (2004) Marijuana and cannabinoid regulation of brain reward circuits. Br J Pharmacol 143(2):227–234. 10.1038/sj.bjp.0705931 PMC1575338 15313883

[CR70] McBurney-Lin J, Vargova G, Garad M, Zagha E, Yang H (2022) The locus coeruleus mediates behavioral flexibility. Cell Rep 41(4):111534. 10.1016/j.celrep.2022.111534 PMC9662304 36288712

[CR71] Meyer PJ, Lovic V, Saunders BT, Yager LM, Flagel SB, Morrow JD, Robinson TE (2012) Quantifying individual variation in the propensity to attribute incentive salience to reward cues. PLoS One 7(6):e38987. 10.1371/journal.pone.0038987 PMC3382216 22761718

[CR72] Meyer HC, Lee FS, Gee DG (2018) The role of the endocannabinoid system and genetic variation in adolescent brain development. Neuropsychopharmacology 43(1):21–33. 10.1038/npp.2017.143 PMC5719094 28685756

[CR73] Moos RH, Moos BS (2006) Rates and predictors of relapse after natural and treated remission from alcohol use disorders. Addiction 101(2):212–222. 10.1111/j.1360-0443.2006.01310.x PMC1976118 16445550

[CR74] Morrow JD, Maren S, Robinson TE (2011) Individual variation in the propensity to attribute incentive salience to an appetitive cue predicts the propensity to attribute motivational salience to an aversive cue. Behav Brain Res 220(1):238–43. 10.1016/j.bbr.2011.02.013 PMC3073129 21316397

[CR75] Murray JE, Bevins RA (2007) Behavioral and neuropharmacological characterization of nicotine as a conditional stimulus. Eur J Pharmacol 561(1–3):91–104. 10.1016/j.ejphar.2007.01.046 PMC1950748 17343849

[CR76] Nautiyal KM, Tanaka KF, Barr MM, Tritschler L, Le Dantec Y, David DJ, Gardier AM, Blanco C, Hen R, Ahmari SE (2015) Distinct circuits underlie the effects of 5-HT1B receptors on aggression and impulsivity. Neuron 86(3):813–26. 10.1016/j.neuron.2015.03.041 PMC4431594 25892302

[CR77] Nonkes LJ, Homberg JR (2013) Perseverative instrumental and Pavlovian responding to conditioned stimuli in serotonin transporter knockout rats. Neurobiol Learn Mem 100:48–55. 10.1016/j.nlm.2012.12.004 23261854

[CR78] Olausson P, Jentsch JD, Taylor JR (2004) Repeated nicotine exposure enhances responding with conditioned reinforcement. Psychopharmacology (Berl) 173(1–2):98–104. 10.1007/s00213-003-1702-9 14727001

[CR79] Oleson EB, Cheer JF (2012) A brain on cannabinoids: the role of dopamine release in reward seeking. Cold Spring Harb Perspect Med. 10.1101/cshperspect.a012229 PMC3405830 22908200

[CR80] Overby PF, Daniels CW, Del Franco A, Goenaga J, Powell GL, Gipson CD, Sanabria F (2018) Effects of nicotine self-administration on incentive salience in male Sprague Dawley rats. Psychopharmacology (Berl) 235(4):1121–1130. 10.1007/s00213-018-4829-4 29374305

[CR81] Palmatier MI, Matteson GL, Black JJ, Liu X, Caggiula AR, Craven L, Donny EC, Sved AF (2007) The reinforcement enhancing effects of nicotine depend on the incentive value of non-drug reinforcers and increase with repeated drug injections. Drug Alcohol Depend 89(1):52–59. 10.1016/j.drugalcdep.2006.11.020 PMC1955684 17240084

[CR82] Palmatier MI, Marks KR, Jones SA, Freeman KS, Wissman KM, Sheppard AB (2013) The effect of nicotine on sign-tracking and goal-tracking in a Pavlovian conditioned approach paradigm in rats. Psychopharmacology 226(2):247–259. 10.1007/s00213-012-2892-9 PMC12989761 23090624

[CR83] Parkinson JA, Dalley JW, Cardinal RN, Bamford A, Fehnert B, Lachenal G, Rudarakanchana N, Halkerston KM, Robbins TW, Everitt BJ (2002) Nucleus accumbens dopamine depletion impairs both acquisition and performance of appetitive Pavlovian approach behaviour: implications for mesoaccumbens dopamine function. Behav Brain Res 137(1–2):149–163. 10.1016/s0166-4328(02)00291-7 12445721

[CR84] Pasquariello KZ, Han M, Unal C, Meyer PJ (2018) Adrenergic manipulation inhibits Pavlovian conditioned approach behaviors. Behav Brain Res 339:278–285. 10.1016/j.bbr.2017.10.037 PMC5729111 29128392

[CR85] Perkins KA, Donny E, Caggiula AR (1999) Sex differences in nicotine effects and self-administration: review of human and animal evidence. Nicotine Tob Res 1(4):301–315. 10.1080/14622299050011431 11072427

[CR86] Perkins KA, Karelitz JL, Boldry MC (2017) Nicotine acutely enhances reinforcement from non-drug rewards in humans. Front Psychiatry 8:65. 10.3389/fpsyt.2017.00065 PMC5410601 28507522

[CR87] Pitchers KK, Phillips KB, Jones JL, Robinson TE, Sarter M (2017) Diverse roads to relapse: a discriminative cue signaling cocaine availability is more effective in renewing cocaine seeking in goal trackers than sign trackers and depends on basal forebrain cholinergic activity. J Neurosci 37(30):7198–7208. 10.1523/jneurosci.0990-17.2017 PMC5546399 28659281

[CR88] Prasad K, de Vries EFJ, Elsinga PH, Dierckx R, van Waarde A (2021) Allosteric interactions between adenosine A(2A) and dopamine D(2) receptors in heteromeric complexes: biochemical and Pharmacological Characteristics, and opportunities for PET imaging. Int J Mol Sci 22(4). 10.3390/ijms22041719 PMC7915359 33572077

[CR89] Prediger RD, Cunha D, C., Takahashi RN (2005) Antagonistic interaction between adenosine A2A and dopamine D2 receptors modulates the social recognition memory in reserpine-treated rats. Behav Pharmacol 16(4):209–218. 10.1097/01.fbp.0000166825.62130.9a 15961960

[CR90] Puig MV, Gulledge AT (2011) Serotonin and prefrontal cortex function: neurons, networks, and circuits. Mol Neurobiol 44(3):449–64. 10.1007/s12035-011-8214-0 PMC3282112 22076606

[CR91] Quarta D, Ferré S, Solinas M, You ZB, Hockemeyer J, Popoli P, Goldberg SR (2004) Opposite modulatory roles for adenosine A1 and A2A receptors on glutamate and dopamine release in the shell of the nucleus accumbens. Effects of chronic caffeine exposure. J Neurochem 88(5):1151–1158. 10.1046/j.1471-4159.2003.02245.x 15009670

[CR92] Ragia G, Manolopoulos VG (2018) From Homer and Hippocrates to modern personalized medicine: is there a role for pharmacoepigenomics in the treatment of alcohol addiction? Pharmacogenomics 19(6):513–516. 10.2217/pgs-2018-0025 29580163

[CR93] Reeves KC, Shah N, Muñoz B, Atwood BK (2022) Opioid receptor-mediated regulation of neurotransmission in the brain. Front Mol Neurosci 15:919773. 10.3389/fnmol.2022.919773 PMC9242007 35782382

[CR94] Robinson TE, Flagel SB (2009) Dissociating the predictive and incentive motivational properties of reward-related cues through the study of individual differences. Biol Psychiatry 65(10):869–873. 10.1016/j.biopsych.2008.09.006 PMC2737368 18930184

[CR95] Rorabaugh JM, Chalermpalanupap T, Botz-Zapp CA, Fu VM, Lembeck NA, Cohen RM, Weinshenker D (2017) Chemogenetic locus coeruleus activation restores reversal learning in a rat model of alzheimer’s disease. Brain 140(11):3023–3038. 10.1093/brain/awx232 PMC5841201 29053824

[CR96] Roughley S, Killcross S (2019) Differential involvement of dopamine receptor subtypes in the acquisition of Pavlovian sign-tracking and goal-tracking responses. Psychopharmacology (Berl) 236(6):1853–1862. 10.1007/s00213-019-5169-8 PMC6602989 30683942

[CR97] Saunders BT, Robinson TE (2012) The role of dopamine in the accumbens core in the expression of Pavlovian-conditioned responses. Eur J Neurosci 36(4):2521–2532. 10.1111/j.1460-9568.2012.08217.x PMC3424374 22780554

[CR98] Saunders BT, Robinson TE (2013) Individual variation in resisting temptation: implications for addiction. Neurosci Biobehav Rev 37(9 Pt A):1955–1975. 10.1016/j.neubiorev.2013.02.008 PMC3732519 23438893

[CR99] Stringfield SJ, Palmatier MI, Boettiger CA, Robinson DL (2017) Orbitofrontal participation in sign- and goal-tracking conditioned responses: effects of nicotine. Neuropharmacology 116:208–223. 10.1016/j.neuropharm.2016.12.020 PMC5385154 28012948

[CR100] Stringfield SJ, Boettiger CA, Robinson DL (2018) Nicotine-enhanced Pavlovian conditioned approach is resistant to omission of expected outcome. Behav Brain Res 343:16–20. 10.1016/j.bbr.2018.01.023 PMC5833299 29407412

[CR101] Stringfield SJ, Madayag AC, Boettiger CA, Robinson DL (2019) Sex differences in nicotine-enhanced Pavlovian conditioned approach in rats. Biol Sex Differ 10(1):37. 10.1186/s13293-019-0244-8 PMC6637589 31315660

[CR102] Tomie A, Grimes KL, Pohorecky LA (2008) Behavioral characteristics and neurobiological substrates shared by Pavlovian sign-tracking and drug abuse. Brain Res Rev 58(1):121–135. 10.1016/j.brainresrev.2007.12.003 PMC2582385 18234349

[CR103] Townsend ES, Amaya KA, Smedley EB, Smith KS (2023) Nucleus accumbens core acetylcholine receptors modulate the balance of flexible and inflexible cue-directed motivation. Sci Rep 13(1):13375. 10.1038/s41598-023-40439-4 PMC10435540 37591961

[CR104] Tseng KY, Molla HM (2025) Cannabinoid CB1 receptor-sensitive neurodevelopmental processes and trajectories. Mol Psychiatry 30(8):3792–3803. 10.1038/s41380-025-03057-2 PMC12240843 40389627

[CR105] Tunstall BJ, Kearns DN (2015) Sign-tracking predicts increased choice of cocaine over food in rats. Behav Brain Res 281:222–228. 10.1016/j.bbr.2014.12.034 PMC4305489 25541036

[CR106] Verplaetse TL, Carretta RF, Struble CA, Pittman B, Roberts W, Zakiniaeiz Y, Peltier MR, McKee SA (2025) Gender differences in alcohol use disorder trends from 2009–2019: an intersectional analysis. Alcohol 123:101–107. 10.1016/j.alcohol.2024.11.003 PMC11871986 39579801

[CR107] Versaggi CL, King CP, Meyer PJ (2016) The tendency to sign-track predicts cue-induced reinstatement during nicotine self-administration, and is enhanced by nicotine but not ethanol. Psychopharmacology (Berl) 233(15–16):2985–2997. 10.1007/s00213-016-4341-7 PMC4935618 27282365

[CR108] Wenzel JM, Oleson EB, Gove WN, Cole AB, Gyawali U, Dantrassy HM, Bluett RJ, Dryanovski DI, Stuber GD, Deisseroth K, Mathur BN, Patel S, Lupica CR, Cheer JF (2018) Phasic dopamine signals in the nucleus accumbens that cause active avoidance require endocannabinoid mobilization in the midbrain. Curr Biol 28(9):1392-1404e.e1395. 10.1016/j.cub.2018.03.037 PMC5940536 29681476

[CR109] Winstanley CA, Dalley JW, Theobald DE, Robbins TW (2004) Fractionating impulsivity: contrasting effects of central 5-HT depletion on different measures of impulsive behavior. Neuropsychopharmacology 29(7):1331–1343. 10.1038/sj.npp.1300434 15054475

[CR110] Yadid G, Gispan I, Lax E (2013) Lateral habenula deep brain stimulation for personalized treatment of drug addiction. Front Hum Neurosci 7:806. 10.3389/fnhum.2013.00806 PMC3860270 24376408

[CR111] Zhu Y, Evans EA, Mooney LJ, Saxon AJ, Kelleghan A, Yoo C, Hser YI (2018) Correlates of long-term opioid abstinence after randomization to methadone versus buprenorphine/naloxone in a multi-site trial. J Neuroimmune Pharmacol 13(4):488–497. 10.1007/s11481-018-9801-x PMC6224303 30094695

